# From monitoring metrics to decision evidence: validating actionability in applied sport science

**DOI:** 10.3389/fspor.2026.1983979

**Published:** 2026-09-30

**Authors:** Cyril Besson, Frédéric Stucky

**Affiliations:** 1 Sports Medicine Center, Swiss Olympic Medical Center, Lausanne University Hospital, Lausanne, Switzerland; 2 Institute of Sport Sciences, University of Lausanne, Lausanne, Switzerland; 3 College of Sports Science and Technology, Mahidol University, Salaya, Thailand; 4 Sports Medicine & Performance Center, Mahidol University, Salaya, Thailand

**Keywords:** actionability, applied sport science, athlete monitoring, dashboards, decision-making, measurement validity, recovery, training load

## Abstract

In high-performance sport, athlete monitoring and management systems now make physiological, biomechanical, and contextual data easier to collect and share than ever before. Yet it remains unclear whether this expansion has produced better decisions or improved athlete-relevant outcomes. Existing frameworks classify monitoring tools, establish standards for verifying and validating biometric technologies for a stated use, and, increasingly, link patterns of monitoring signals to candidate coaching actions. These advances are necessary, but they do not establish whether valid monitoring information influences decisions or improves the outcomes those decisions are intended to affect. In this Perspective, we argue that actionability should be treated as a claim about decision utility rather than as an inherent property of a metric or monitoring system. We distinguish two levels of evaluation: decision impact, whether monitoring information influences the decision that would otherwise have been made; and outcome evidence, whether the resulting decision improves or protects athlete-relevant outcomes. Practical value also depends on whether a pathway is implemented as intended and whether its benefits justify the burden of implementation. To support evaluation in applied settings, we outline feasible approaches ranging from descriptive audits and decision-impact studies to pragmatic outcome designs. Although our primary focus is high-performance sport, the same decision-evidence logic may extend to health, rehabilitation, and amateur sport. Ultimately, monitoring should be judged by the strength of evidence linking its use to decisions that serve the athlete's best interest.

## From more data to better decisions

In high-performance sport, athlete monitoring and management systems now make physiological, biomechanical, and contextual data easier to collect, organize, and share than was possible two decades ago (1–3). In principle, these systems are used to inform decisions aimed at supporting performance while safeguarding athlete health and wellbeing. Yet, a more consequential question remains unresolved: has more data produced better decisions and improved athlete-relevant outcomes?

Existing work has clarified several important aspects of how monitoring should be used. Practical frameworks describe how monitoring data may be interpreted and applied (4), while appropriate classification of monitoring tools has been emphasized as a prerequisite for interpreting their outputs (5). Other studies have focused more directly on the role of monitoring in practitioner decision-making, framing monitoring as a decision-support process rather than a standalone determinant of performance outcomes (6, 7). Whether monitoring information actually influences decisions remains unclear. In applied settings, feedback processes and perceived practical impact varied substantially (3), and monitoring data may function as communication or status information without a tested basis for influencing decisions (8). Further downstream, evidence that monitoring-informed practice improves athlete-relevant outcomes also remains scarce (7).

These developments clarify important parts of the monitoring process, but they do not establish actionability. Indeed, a monitoring signal can be measurable, reliable, physiologically plausible, and appropriately classified, yet still fail to inform a predefined decision pathway intended to improve or protect an athlete-relevant outcome. In this Perspective, we argue that actionability should be treated as a claim about decision utility rather than as an inherent property of a metric or monitoring system. We examine what current monitoring frameworks establish and what they leave untested, before outlining the evidence needed to close that gap. Although our primary focus is high-performance sport, the same decision-evidence logic extends to health, rehabilitation, and amateur sport, where the relevant outcomes and acceptable trade-offs may differ.

## What existing frameworks solve and what they leave open

Formalized frameworks have started to clarify what different monitoring tools are designed to measure. Buchheit and Hader, for instance, proposed a quadrant-based approach that separates load from response or adaptation, and metabolic from neuromuscular domains, as a way to reduce the risk of using tools outside their physiological purpose (5). This matters because classification errors are common. For instance, heart rate variability (HRV) is not internal training load; it is a response-related marker mainly shaped by autonomic regulation, fitness, fatigue, sleep, illness, and training phase (9, 10). Similarly, GPS metrics are external movement outputs, not direct measures of internal load or tissue-level neuromuscular response (5, 11). A practitioner who manages load using a marker that reflects something else is building a decision pathway on the wrong mechanism from the start.

Biomarker and digital-health science frameworks have added another essential layer. The V3 framework for biometric monitoring technologies distinguishes verification, analytical validation, and clinical validation, together establishing whether a technology is fit-for-purpose for a stated context of use (12) (Figure 1, Layer A). Within this validation layer, measurement-error frameworks remain essential for determining whether an observed change in a monitoring signal is likely to exceed measurement noise (13, 14). More recently, the rapid expansion of sensor technologies and artificial intelligence (AI) has reinforced the need for standardized evaluation before their outputs are used to guide practice (15). AI-enabled consumer-grade wearables have also been classified according to their progression from automated data collection to automated insights, recommendations, and individualized exercise decisions (16).

**Figure 1:**
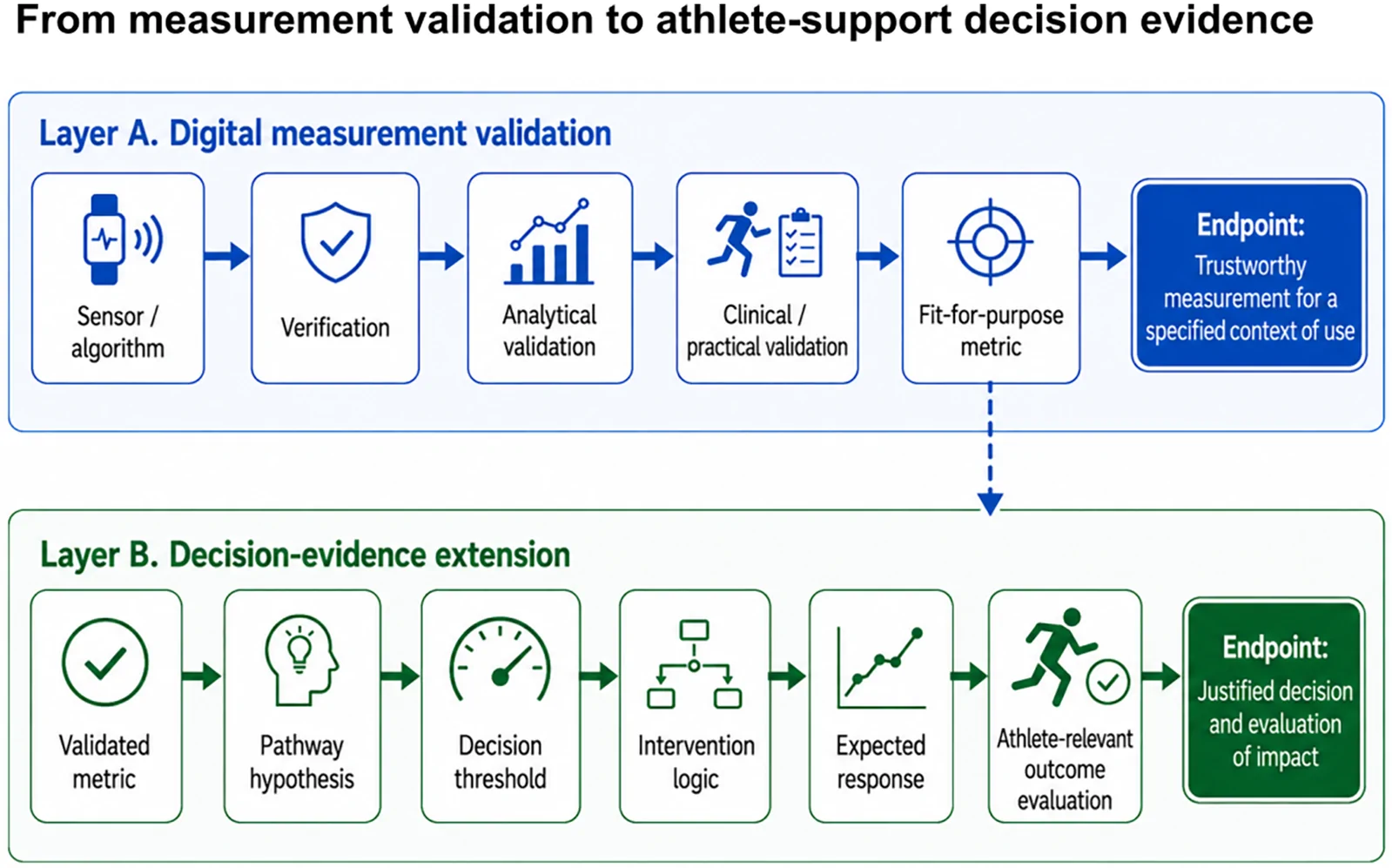
Conceptual framework linking digital measurement validation to decision evidence. Verification, analytical validation, and clinical or practical validation establish whether a monitoring output is trustworthy for a specified context of use. The decision-evidence layer then asks whether the validated output supports a defined decision pathway and improves or at least protects athlete-relevant outcomes.

A further body of work has moved toward structuring monitoring data explicitly for practitioner decision-making. Data-analytics frameworks in elite sport distinguish descriptive, diagnostic, predictive, and prescriptive analytics, with prescriptive analytics requiring a stronger link between information and action (17). Others examined traffic-light systems in team-sport monitoring and noted that their outputs are difficult to validate against injury or athlete-relevant outcomes, describing such systems as explanatory rather than predictive (18). These approaches help to organize and communicate monitoring information, but they do not necessarily define what action should follow. A second group of frameworks goes further by linking monitoring profiles to candidate actions. The LOAD-R model links training load, athlete response, and an adaptive-state classification to a defined set of coaching actions and proposes testable predictions for evaluating the sequence (19). A complementary framework combines the Minimal-Adequate-Accurate principle (20) for tool selection with individualized thresholds and a quadrant-based visualization linking load, neuromuscular, and wellbeing signals to specific coaching actions (7). This is conceptually aligned with the load-response and quadrant logic of Buchheit and Hader (5) but should be viewed as complementary rather than as a direct extension.

These frameworks are valuable because they improve classification, technical validation, and decision architecture. However, they do not, by themselves, establish decision utility. A framework may show what a signal reflects, whether it can be trusted, and how it might be combined into an action, without demonstrating that the resulting pathway significantly influences athlete-support decisions or improves athlete-relevant outcomes. Goldsack et al. make a similar distinction by noting that “clinical utility” is a further step beyond V3 (12), and biomarker science has debated the same issue for years: a test can be analytically and clinically valid without being useful enough to guide action (21).

## Actionability is a decision-utility claim

These limitations share the same root cause: measurement itself is being treated as decision-support evidence, when actionability is in fact its own empirical claim. Establishing that claim requires first defining the decision pathway: what information is considered, under what conditions, what action may follow, and what response is expected. Second, that pathway must be evaluated at two related but distinct levels: *decision impact*, whether the monitoring information influences the decision that would otherwise have been made; and *outcome evidence*, whether the resulting decision improves an athlete-relevant outcome (Figure 1, Layer B). Even when the information does change a decision, this does not establish that the resulting action improves the outcome it is intended to influence (22). This distinction echoes a hierarchy long used in health technology assessment, in which evidence that a test changes clinical decisions is necessary but not sufficient to establish that it improves patient outcomes (23).

Two examples illustrate why decision impact and outcome evidence must be separated. A cluster randomized trial of Acute:Chronic Workload Ratio-guided load management in 482 elite youth footballers found no reduction in the prevalence of health issues over a 10-month season (24). Coaches’ adherence to the protocol was assessed only through a brief post-study questionnaire rather than records of how training decisions were influenced by monitoring information. The null result therefore cannot distinguish an ineffective decision rule from a decision-impact failure, since it remains unclear whether the pathway was followed as intended. Heart rate variability-guided training illustrates the complementary case: because training intensity is directly prescribed by the day's HRV reading, decision impact is fixed by protocol, which allows testing outcome evidence in isolation. Meta-analyses using between-group comparisons show mixed or modest outcomes, with some physiological gains, but no consistent performance advantage (25, 26).

In these examples, even when decision impact can be assumed, the available outcome evidence has been modest; where it cannot, the two remain entangled. Neither case matches the confidence with which such approaches are commonly adopted and advertised. Actionability does not require intervention whenever a signal changes, but it does require that action or deliberate non-action follows an explicit and defensible pathway, a distinction already established in clinical decision-support research (27). Additionally, decision impact and outcome evidence can only be attributed to a pathway if that specific pathway was implemented as intended. This requires documenting whether the athlete-support team (the practitioners involved in athlete care and performance support, including coaches, sport scientists, medical staff, nutritionists, psychologists, and other relevant staff) followed, overrode, or adapted the decision rule. For subjective monitoring, a further concern is whether the input itself is trustworthy, since athletes may withhold honest answers when they distrust how the information will be used (28). If either side did not follow the pathway as intended, a study's result cannot be attributed to the decision rule itself.

A separate question is whether the pathway is worth running at all. Even one that changes decisions and improves outcomes can still impose a burden that outweighs its benefit (29). The same pattern is well documented in medicine: treatment burden drives poor adherence and worse outcomes in chronic disease care, regardless of how sound the regimen is (30). Decision impact and outcome evidence are therefore necessary but not sufficient. Clinical guideline frameworks already separate evidence strength from a treatment's acceptability, feasibility, and cost, since a well-evidenced option can still be the wrong one to use (31). Sometimes the right call is to reduce or drop a monitoring procedure once its burden outweighs its value.

The pathway, rather than the individual metric, is therefore the relevant unit of evaluation. In practice, decisions rarely depend on one signal in isolation. This is consistent with broader “fine-tuning” approaches in which monitoring information is adapted to the athlete, context, and training phase rather than applied through a single generic threshold (32). Similarly, decisions to modify training may integrate training load, injury history, recovery status, competition demands, and practitioner judgment rather than follow a single threshold mechanically (8).

Such contextual and multivariable decision-making by practitioners does not prevent formal evaluation. Evidence-based medicine has long combined research evidence and clinical judgment (33), and decision rules that incorporate several signals and let the clinician override them are still tested for accuracy and impact (34). A multivariable pathway can still be evaluated if its decision logic is explicit enough to be applied consistently and compared with an alternative approach. Initial studies may begin with relatively simple pathways containing few contextual branches before extending the same logic to richer and more context-dependent decisions. How this is operationalized will vary across sports, competitive levels, staffing models, and decision contexts. Later in this article, we outline candidate study designs that could generate decision impact and outcome evidence. Before doing so, it is worth considering why this evidence is not already routinely produced.

## Where the actionability gap appears in practice

The gap between validating a measurement and validating a decision pathway may seem obvious in theory, but it is often blurred in practice in at least two common ways. The first is metric accumulation: as monitoring tools become more available and affordable (1, 2), a common risk is to build monitoring systems around variables that are convenient to collect or visually persuasive, rather than around a defined performance- or health-related question. Passing technical or analytical validation does not resolve this. Validation establishes whether a signal is trustworthy for a specified measurement purpose; actionability depends on whether the signal is embedded in a decision pathway and whether using that pathway changes decisions and/or outcomes (12, 21, 23).

Along the same lines, monitoring dashboards may create a false impression of actionability: by converting heterogeneous and uncertain signals into a clean score, color, label, or risk flag, they can appear to provide decision support even when the next action, uncertainty, cost of error, and expected response have not been defined. A dashboard can summarize information and make complex data easier to discuss, but the key question is not how clearly it displays data, it is what claim is attached to its outputs. Recent quadrant-based visualizations illustrate this well: Rebelo et al. map combinations of load, neuromuscular performance, and wellbeing onto labeled coaching actions such as “train to adapt” or “investigate fatigue” (7). Tools of this kind give the athlete-support team a shared, physiologically grounded structure for a decision that would otherwise be made implicitly, and their explicitness is also what makes them suitable candidates for the kind of decision-impact and outcome evaluation described below. An “at risk” flag only becomes decision support once its decision context is defined: what actions are available, how uncertain the signal is, the likely cost of a false positive or false negative, and what response is expected after action. Monitoring data is not always interpreted or applied as intended, with some athletes and coaches receiving little or no meaningful follow-up (3, 4). The next step is to make the evidentiary status of that application explicit. A dashboard without a defined next action may still be useful for communication, but it should not be presented as a validated decision-support system.

## Why decision evidence remains scarce

The production of decision evidence in athlete monitoring is shaped by commercial and academic incentives that do not always favor its generation or dissemination. Commercial incentives favor speed to market over validated decision impact. Consumer wearable manufacturers release new products at a pace that outstrips the academic validation cycle. The resulting gap is substantial even at the more basic level of measurement accuracy: a review found that of 310 consumer wearables released since 2003, only around 11% had been validated for even one biometric outcome, and only 3.5% of the validation studies needed for a complete picture of device accuracy have been conducted (2). Decision-impact evidence, a further and more demanding step, is scarcer still. This pressure also reaches athlete-support teams directly, who may face marketing that presents a tool's technical sophistication as though it already implied decision utility. These pressures do not invalidate innovation, but they can make weakly validated metrics appear more mature than they are.

Publishing incentives add further complication. Open-access publishing has improved accessibility, but article processing charges remain a cost barrier, and article volume is still commonly used as a marker of productivity (35, 36), creating a structural disadvantage for studies that track decisions or outcomes over time and therefore take longer to complete than cross-sectional validation studies. Sport and exercise scientists have separately called for more transparent research practices (37), but competitive confidentiality remains a major obstacle. For example, in a systematic review of sports injury prediction models, none of 30 studies made their underlying data or code available (38), a gap attributed to teams protecting proprietary data, journal practices, and limited training in open-science methods (39). This does not necessarily mean that evaluation is absent in practice; some may remain internal to teams or commercial providers and therefore never enter the scientific evidence base. Applied sport science has no regulatory gate equivalent to biomarker qualification, so journal standards, reviewer expectations, and voluntary reporting frameworks may be the most realistic points of control. The core risk is practical authority without sufficient evidence. A metric may be easy to collect, visually compelling, and commercially promoted before the assumptions behind its use have been tested. A dashboard can then appear to offer decision support when it is mainly displaying organized information. Journals and reviewers should distinguish clearly between studies showing that a metric can be collected, studies showing that it is associated with an outcome, and studies showing that decisions built on it improve athlete-relevant outcomes.

## Toward a research agenda for validating actionability

The purpose of this Perspective is not to prescribe a single method for validating actionability, but to identify the evidence needed to determine whether monitoring information influences decisions and whether those decisions ultimately improve outcomes in the athlete's best interest. With the decision pathway defined, evaluation can proceed from relatively simple pathways to richer, more context-dependent ones.

Consider a practical example, consistent with the convergence logic underlying recent decision-architecture frameworks (7, 19): if a player's session distance exceeds their individual 4-week rolling average by more than 20% and their next-morning perceived recovery score falls below their typical range, reduce next-day training volume by one planned tier; if only one signal is present, maintain the plan. This rule specifies a convergence condition, a contextual check, and an action, making it suitable for evaluation. Descriptive audits, potentially incorporating systematic behavioral observation as used in other sport contexts (40), could establish whether such pathways are used as intended in practice. In our example, this would involve documenting how often the two signals converge, whether the prescribed training reduction is implemented or overridden, and whether the monitoring output provides information beyond what athletes or the athlete-support team already know. The same audits could also check if the inputs themselves are trustworthy, for example by identifying identical responses across clearly different circumstances, improbably fast completion times, or missing responses during demanding periods. Audit-and-feedback approaches are particularly relevant because they compare practice against an explicit standard and are more effective when linked to a defined target and action plan (41).

Decision-impact studies could then test whether access to the monitoring output changes practice. In medicine, such designs have compared decisions made with and without access to an adjunctive diagnostic report, while keeping the standard assessment unchanged in both conditions (22). Analogous studies in sport could use athlete-support team meetings or simulated/retrospective cases to test whether monitoring information changes training prescription, recovery planning, medical screening, or athlete communication (in our example, whether athlete-support teams reviewing past cases would have reduced training volume differently when the converging signals were shown or hidden).

Outcome studies would address the further question of whether those changed decisions improve athlete-relevant outcomes, and whether those effects remain favorable when relevant consequences for performance and health are considered together. This is difficult because the counterfactual outcome of an individual decision is never directly observed: when an athlete is withheld and remains healthy, it is unknown what would have occurred had they trained or competed (8). Pragmatic designs can provide comparison periods or groups without requiring conventional large-sample trials. Stepped-wedge designs, for example, could introduce a monitoring pathway progressively across squads, allowing periods with and without the pathway to be compared before it is eventually adopted by all groups (42). Repeated case series could examine the effects of the same decision pathway across multiple athletes or periods, while N-of-1 designs could compare outcomes within the same athlete under different monitoring conditions (43, 44). These approaches are illustrative rather than prescriptive. Most are established in neighboring fields but remain underused for evaluating monitoring-based decisions in sport. The aim is not to eliminate uncertainty, but to ensure that claims of actionability do not exceed the evidence supporting them.

## Stronger science, not less science

Our argument is intentionally critical, but not anti-technology or anti-innovation. Existing work has already called for better classification of monitoring tools (5) and more standardized evaluation of sensors and AI (15). The next step is to determine when valid measurement becomes useful decision support. If this argument has one practical priority, it is that decision-impact and outcome evidence should be weighed at least as heavily as technical or analytical sophistication. A simple metric linked to demonstrated decision impact may be more useful than a complex metric that lacks it, echoing an earlier concern about sport science's appetite for technological complexity (45).

There is reason to expect this approach to be useful. In clinical medicine, decision support systems that provided an explicit actionable recommendation succeeded significantly more often than systems that only flagged an assessment, and systems requiring a documented reason for not following the recommendation succeeded more often still (27). This evidence comes from medicine, not sport, and even there, roughly half of related trials met real barriers such as alert fatigue and poor workflow fit. The point is not that the same effects are already established in sport, but that the underlying decision-support logic has empirical support elsewhere.

This call is realistic in parts. Descriptive audits and decision-impact studies are feasible now: they require little additional infrastructure, only the decision to look. Outcome studies are more difficult. Elite sport offers small samples, rare events, and many uncontrolled variables changing at once; researchers in this field have reached the same conclusion, noting that the needed randomized controlled trial of load management “will be a difficult study to execute at any level of sport” (46). Sport science also does not carry medicine's public-health stakes, so a regulatory apparatus proportional to those stakes is neither realistic nor necessary here. For both reasons, the designs proposed above are deliberately lighter weight.

Our argument also has limitations. Some claims, especially those concerning academic, commercial, and publishing incentives, are interpretive and should be tested empirically. Nor do we propose a replacement for existing validation frameworks; we build on them.

Before monitoring systems are presented as decision-support tools, the assumptions linking signals, pathways, decisions, and outcomes should be stated clearly and tested where possible. The implication is not that applied sport science needs less technology, but that technology should be grounded in physiology, context, and explicit decision logic. A monitoring signal becomes actionable only when it is embedded in a defined pathway that influences decisions and improves or protects athlete-relevant outcomes. The practical value of a monitoring system should ultimately be judged by whether it serves the athlete's best interest, and whether its benefits outweigh the burden of implementation.

## Data Availability

The original contributions presented in the study are included in the article/Supplementary Material, further inquiries can be directed to the corresponding authors.
